# Lifestyle-related effects of the web-based Kanker Nazorg Wijzer (Cancer Aftercare Guide) intervention for cancer survivors: a randomized controlled trial

**DOI:** 10.1007/s11764-016-0535-6

**Published:** 2016-03-17

**Authors:** Iris M. Kanera, Catherine A. W. Bolman, Roy A. Willems, Ilse Mesters, Lilian Lechner

**Affiliations:** 1 Faculty of Psychology and Educational Sciences, Open University of the Netherlands, P. O. Box 2960, 6401 DL Heerlen, The Netherlands; 2 CAPHRI School for Public Health and Primary Care, Optimizing Patient Care, Maastricht University, P.O. Box 616, 6200 MD Maastricht, The Netherlands

**Keywords:** Cancer survivorship, Physical activity, Nutrition, Smoking, eHealth, Computer tailoring

## Abstract

**Purpose:**

The web-based *Kanker Nazorg Wijzer* (Cancer Aftercare Guide) responds to the needs of cancer survivors and oncology care providers to improve the counseling related to self-management of lifestyle and psychosocial challenges. In present study, overall intervention effects and the effects of using specific components were evaluated on vegetable, fruit, whole grain bread, and fish consumption, physical activity (PA), and smoking behavior.

**Methods:**

Cancer survivors from 21 Dutch hospitals were recruited for a randomized controlled trial (*N* = 432). Intervention effects after 6 months were evaluated using multilevel linear regression analysis (complete cases and intention-to-treat). By conducting moderation analyses, additional effects of following the behavior-related modules were explored. The false discovery rate correction was applied to account for multiple testing.

**Results:**

After 6 months, 409 participants completed follow-up (dropout = 11.5 %). Indications were found that access to the intervention may result in increases of moderate PA and vegetable intake. The moderate PA increase was meaningful: 74.74 min p/w higher increase in the intervention condition. Effect sizes of moderate PA (*d* = .25) and vegetable (*d* = .37) consumption were comparable to prior effective interventions. Visiting behavior-related modules affected moderate PA, fruit, and fish consumption. However, after correction for multiple testing, significances expired. No significant intervention effect was found on smoking behavior due to low numbers of smokers.

**Implications for Cancer Survivors:**

Although the effectiveness was only shown only to a limited extend, this study provided several indications that this theory-based, comprehensive, and personalized eHealth intervention provides valuable content to complement usual cancer aftercare.

## Introduction

**Fig. 1:**
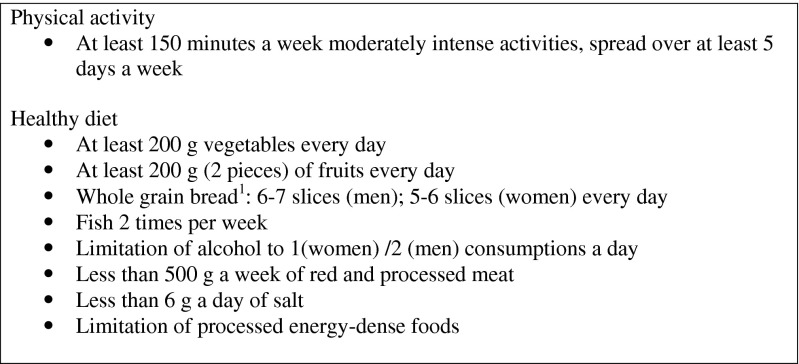
Lifestyle recommendations for cancer survivors used in the KNW intervention. Adapted from “Food, Nutrition, Physical Activity, and the Prevention of Cancer: a Global Perspective.” by WCRF/AICR, 2009, and “American Cancer Society Guidelines on Nutrition and Physical Activity for Cancer Prevention. Reducing the Risk of Cancer With Healthy Food Choices and Physical Activity” by Kushi et al., 2012. Not all of the recommendation of the WCRF/AICR and the American Cancer Society are displayed. ^1^ For persons aged 18–70 years

Healthy lifestyle behaviors have proven to be highly beneficial for cancer survivors in improving recovery and quality of life and lowering the risk of cancer recurrence and comorbidities [1–6]. As a result, comprehensive lifestyle recommendations have been developed by the World Cancer Research Fund/American Institute for Cancer Research (WCRF/AICR) and the American Cancer Society [7–9]. The recommendations with regard to physical activity (PA) and dietary behavior are displayed in Fig. 1. In addition, it is advised to refrain from smoking [5, 6, 10, 11]. Nonetheless, and despite the beneficial effects, only about 30–47 % of cancer survivors follow the PA recommendations, about 15–34 % follow the vegetable and fruit recommendations, and about 7.8–20.8 % continue to smoke [12–17]. In turn, cancer survivors have indicated unmet needs in psychosocial and physical domains, including the need for specific and evidence-based information and support to build up PA, to improve their diet, and to quit smoking. These needs have been stressed by oncology care providers as well [18–24]. Moreover, oncologists have expressed a lack of time and expertise as barriers to giving multiple lifestyle behavior advice [24–26]. Consequently, health promotion initiatives should respond to these needs and to the problems of care professionals who are serving a growing number of cancer survivors with restricted time, knowledge, and counseling skills [27–30].

An increasing number of cancer survivors search the Internet for health-related information [31, 32]. Web-based interventions have a wide reach and can be used at any time, any place, and at an individual’s own pace. They might be also less costly than face-to-face interventions [33]. Additionally, web-delivered interventions can effectively provide personalized information by means of computer tailoring (CT), a proven effective method in health behavior change interventions [34–40].

We developed and evaluated a web-based, CT intervention for cancer survivors, named *Kanker Nazorg Wijzer* (Cancer Aftercare Guide, KNW) which aims to complement existing face-to-face aftercare. The detailed research protocol has been described earlier by Willems et al. [41]. The comprehensive content of the KNW covers a combination of multiple lifestyle issues and psychosocial elements, provided through eight specific modules. The lifestyle components of this program operationalized through the modules Physical Activity, Diet, and Smoking are based on assumptions of the Integrated Model for Change (I-Change Model) [42] in which ideas of social-cognitive theories are integrated [43, 44]. Furthermore, change methods derived from of the self-regulation theory [45] were applied. Previous research has demonstrated that interventions targeting cancer survivors’ behavior change, such as PA and dieting are effective when these incorporate social-cognitive theories [46, 47]. The theoretical models explain behavior change as a dynamic process with a series of awareness, initiation, and maintenance phases that are influenced by pre-motivational (awareness and knowledge), motivational (intention, attitude, self-efficacy), and post-motivational determinants (goal setting, action, and coping planning) [48–50]. The theories assume that a continuous process of self-regulation is facilitated through the application of behavior change strategies such as goal setting, action and coping planning, monitoring, evaluating plans, and refining goals when necessary [51, 52]. In addition, the contribution of the determinants to change can differ from one behavior to another and from one person to another [17, 43]. Consequently, behavior change interventions need to be tailored to the specific behavior, individual determinants, and motivational phases [17, 53]. During the development of the KNW, these aspects were taken into account by tailoring the provided information to relevant determinants.

Only few theory-based studies have been conducted which have investigated the effect of web-based interventions aimed at lifestyle outcomes in cancer survivors [54, 55]. Increases in PA and mixed results in diet change have been reported in web-based interventions for cancer survivors using behavior change strategies such as action planning, problem solving, decision-making, and tailoring [56, 57]. Moreover, a usability study revealed that a web-based CT intervention for breast cancer survivors based on the Theory of Planned Behavior and the Transtheoretical Model was well accepted and perceived as interesting, attractive, comprehensible, and credible [58]. Besides that, a web-based smoking cessation intervention among cancer survivors, based on social cognitive theories and tailored to stage of readiness, yielded equivalent levels of success compared to an intervention delivered by telephone [59]. Thus, current evidence is limited but promising concerning the effects of theory grounded, web-based computer-tailored interventions on (multiple) lifestyle behaviors for cancer survivors.

In the present study, we assessed the effects of the KNW on lifestyle outcomes (PA, diet, and smoking) 6 months after getting access to the intervention, among cancer survivors who recently completed primary cancer treatment. First, we assessed whether having access to the KNW may improve PA, diet behavior (fruit, vegetable, whole grain bread, and fish consumption), and can lead to a higher rate of quitters among smokers in comparison to a usual care control group. Second, we explored the effects of following the module Diet on diet outcomes specifically and the effects of following the module PA on PA outcomes.

## Methods

### Trial design

A randomized controlled trial was conducted to reveal effects between participants assigned to the intervention condition (IC) or the usual care control condition (UC). Randomized allocation (ratio of 1:1) was automatically performed by means of a digital randomizer after centralized registration of participants [60]. Ethical approval for this trial (Dutch Trial Register NTR3375) was obtained from the Medical Research Ethics Committee (MERC) Zuyderland-Zuyd (NL41445.096.12, 12-T-115). After approval, the MERC’s and the board of directors of each hospital endorsed the execution of the study.

### Participants

Dutch speaking individuals aged 18 years or older, who have been diagnosed with any type of cancer, and who have completed primary treatment (surgery, chemo-, or radiation therapy) at least 4 weeks and up to 56 weeks prior to initial participation^1^ with no sign of recurrence at the last control visit were eligible to be included in this study. Cancer survivors with severe medical, psychiatric, or cognitive disorders were excluded.

### Procedure

Of the 45 Dutch hospitals approached, 22 hospitals agreed to participate. Medical staff from 21 hospitals recruited eligible participants from November 2013 to June 2014. Unfortunately, one hospital did not include any patients. Medical staff of various outpatient clinics (internal medicine, oncology, gynecology, urology, and breast cancer care) assessed eligibility during the medical consultations or by reviewing patient files. Gender, age, type of cancer, type of treatment, and the termination date of primary cancer treatment were registered for all approached cancer survivors. Eligible cancer survivors received an information package, in person or by post, including comprehensive trial information, a general information brochure about scientific research [61], an informed consent form, a short log-in instruction guide, a storage card with contact details, and personal login codes to the KNW website. Consenting cancer survivors were asked to return the signed informed consent form to the researchers in an enclosed, pre-paid envelope. The participants received a reminder letter after 2 weeks. At the first login to the KNW, participants were automatically randomized to one of the two study conditions and the computer program directly provided information about their allocation. Data from participants who did not return the informed consent forms were excluded from analysis. After randomization, participants were invited to fill out the online self-report baseline questionnaire. Online follow-up measurements were conducted after 3, 6, and 12 months. The IC received access to the KNW throughout the 6 months after completing the baseline assessment while the UC had access to the KNW after completing the 12 months measurement.

### Intervention

A detailed description of the KNW intervention is reported elsewhere [41]. The KNW (http://www.kankernazorgwijzer.nl) is a systematically developed, theory-grounded, web-based intervention aiming to enhance quality of life among early cancer survivors by promoting positive lifestyle changes (i.e., sufficient PA, healthy diet, and smoking cessation) and by providing psychosocial support in the area of fatigue, anxiety and depression, relational problems, and return to work. Each separate topic is integrated in one of the eight KNW modules.

After completing the baseline assessment, participants (IC) received personalized advice on which modules could be most meaningful for them. This advice was based on the responses to the baseline assessment (for detailed information, see Willems et al. [41]). Nevertheless, the program allows the user to make a free selection of all modules based on personal needs and interest.

Technically, the KNW is a fully automated expert system containing an extensive pre-programmed message library that operates without human involvement. By means of CT, individual answers to the baseline assessment are automatically evaluated, and the corresponding messages and intervention fragments from the pre-programmed library are selected and combined using if-then algorithms. Consequently, personalized information is generated [62]. The information within the KNW is tailored to personal characteristics (gender, age, marital status, children, educational level), cancer-related issues (type of cancer, type, and number of comorbidities), motivational determinants (attitude, self-efficacy, and intention), and current behavior (e.g., lifestyle).

Concerning the content of the KNW, seven out of the eight modules are self-management modules and configured to target the specific needs associated with the relevant topic. The eighth module provides general information about the most common residual problems.

The main target of the module PA was to increase PA, during, for example commutes, daily living activities, leisure time, and sports. In the module Diet, the emphasis is placed on increasing healthy eating behaviors through fruit, vegetable, whole grains, and fish consumption. The participants were encouraged to set one or two goals concerning these food groups. Promoting the consumption of healthy food might be a more positive way to achieve changes in diet than by focusing on omission of unhealthy food. More healthy food choices could lead to fewer unhealthy choices. High-fiber diets are, for example, commonly low in fat [63]. However, all dietary recommendations, including the limitation of red meat, fat, sugar, salt, and alcohol consumption, were presented within the module Diet (Fig. 1). The goal of the Smoking module was to support smokers in refraining from smoking. In addition, support was also provided to former smokers to prevent relapse.

Throughout KNW, principles of Problem Solving Therapy have been applied to encourage self-management [64]. Within the lifestyle modules, motivational determinants were addressed based on social-cognitive theories, e.g., the I-Change Model, and self-regulation strategies were applied [42–45]. Used behavior change strategies were consciousness-raising by pointing out the discrepancy between current behavior and recommendations, identifying pros and cons, identifying barriers and providing solutions, persuasive communication, self-monitoring, social modeling, goal setting, action, and coping planning [37, 38, 48, 50]. When visiting a lifestyle module, participants were made specifically aware of their own behavior in relation to the norms. Detailed and personalized feedback targeting attitudes, social support, self-efficacy, barriers, and intentions toward behavior change was provided. Text, photos, videos of fellow survivors and specialists, and hyperlinks to other sources of information were used for this purpose. In addition, the respondents were encouraged to set goals for PA and diet, and smokers were encouraged to set a smoking cessation date. Following this, detailed examples of action and coping plans were provided to help prepare the behavior change [48]. After 4 weeks, participants were invited to evaluate their behavior and encouraged to continue applying the previously provided self-regulation strategies. Furthermore, use of the KNW forum was suggested for interaction with peer cancer survivors and social support.

To encourage the use of KNW, several e-mail reminders and prompts were sent automatically with a direct link to the KNW, for example to invite participants to complete questionnaires or visit modules. Furthermore, additional information was provided by launching monthly news items linked to visiting the website. The KNW was applied without major adjustments, bugs, or downtimes after the trial commencement. Hyperlinks to other websites were updated when needed.

### Outcome measures

#### Physical activity

The validated self-report Short Questionnaire to Assess Health Enhancing Physical Activity (SQUASH) was applied at baseline and at the 6-month follow-up [65–67]. Physical activity was determined based on 11 items including activities during commuting (walking, cycling), leisure time (walking, cycling, gardening, odd jobs), sports (light, moderate, vigorous), household tasks (light work, intense work), and work (light work, intense work). The number of days a week, the average number of minutes a day, and the intensity (light, moderate, vigorous) were rated for all activities. The average weekly minutes of PA were calculated by multiplying the number of days per week with the number of minutes per day, categorized into three categories: “light PA,” “moderate PA,” and “vigorous PA.” In the present study, the outcome measures for PA were “weekly minutes light PA,” “weekly minutes moderate PA,” and “weekly minutes vigorous PA.” One further item was included assessing the number of weekly days with at least the recommended amount of PA by asking: “On how many days a week are you moderately physically active for at least 30 min (e.g., cycling, brisk walking, household, gardening, sports, or other activities)?” [37]. Prior studies supported the reliability and validity of single-item self-report measures for PA [68, 69]. Moreover, reliability and validity of the SQUASH were confirmed in previous research among patient populations [70, 71].

#### Dietary behavior

For assessing vegetable, fruit, whole grain bread, and fish consumption, 8 items of the Dutch Standard Questionnaire on Food Consumption were used at baseline and after 6 months [72]. The number of days per week when products are consumed were asked for each of the food categories (e.g., “How many days a week do you eat fruit?”), ranging from 0 to 7. In addition, the number of servings per day was assessed for fruit (one serving is equal to 100 g), vegetables (one tablespoon is equal to 50 g), whole grain bread (slices), and fish (servings). The mean daily consumption was calculated by multiplying the number of days by the amount of servings and dividing this by 7 days a week. For fish, servings per week were measured, and the mean weekly consumption was calculated. Outcome measures for dietary behavior in the present study were vegetable consumption in grams per day (g p/d), fruit consumption in servings p/d, whole grain bread consumption in slices p/d, and fish consumption in servings p/w. Previous research support the reliability and validity of a similar food frequency questionnaire assessing vegetables en fruit among women [73].

#### Smoking behavior

Standardized questions from Dutch Measuring Instruments for Research on Smoking and Smoking Cessation were used to assess smoking behavior [74]. Based on a combination of three items, current and former smoking behavior was measured at baseline (i.e., “Do you currently smoke”; “Did you smoke in the past?”; “How long ago did you stop smoking?”) and categorized into never-smokers, former smokers, and current smokers. At follow-up, 4 items were used to assess smoking behavior. This behavior was categorized into “current smokers” (“I still smoke, and I did not attempt to quit”), “never-smokers” (“I never smoked, I’m a non- smoker”), and “former smokers” (“I have not smoked a single puff since quitting”). It was also measured whether participants who smoked at baseline quit smoking by means of the standardized 7-day point prevalence abstinence question (“Have you smoked one or more cigarettes/cigars/pipes during the past seven days”) [75, 76]. To identify the intervention effect after 6 months on smoking behavior (yes = 0, no = 1), only the subsample of participants who were smokers at baseline was analyzed.

#### Other relevant measures

Background information was collected at baseline using standard questions on age, gender, marital status (“with partner”: married, cohabiting partners; “without partner”: single, divorced, widowed), education level (“low”: lower vocational education, medium general secondary education; “medium”: secondary vocational education, higher general secondary education; “high”: higher vocational education, university education), income level (“below average”: <€1800 per month; “average”: >€1800 and <€2200 per month; “above average”: >€2200 per month), employment status (“working”: self-employed, in paid employment; “not working”: unemployed, retired, unable to work), type of cancer, type of treatment, time since completion of primary treatment, aftercare, comorbidities, length and weight (body mass index [BMI]). Although other variables were also assessed, these were not used for the current study. “Following specific modules” and the “number of weeks since first login” were derived from program logging data.

### Sample size

Sample size calculation revealed that each intervention condition needed to contain 144 participants (effect size = .30; one-sided *α* = 0.05; *β* = 0.2; power = 80 %); intra-class correlation coefficient (ICC) = 0.005). With an expected dropout of some 20–23 %, the required sample size was *N* = 376 (188 per condition) at baseline.

### Statistical analyses

Preparatory and descriptive analyses were conducted using SPSS 22, and for calculation of the intervention effects, STATA version 13.1 was applied. The dataset was assessed for outliers and aberrant measurement data.

Baseline differences between IC and UC concerning lifestyle behaviors, demographic and cancer-related characteristics were examined using independent *t* tests and chi-square tests. Selective dropout was assessed by applying logistic regression analysis with dropout as outcome variable (0 = no; 1 = yes) and group assignment and baseline characteristics as predictive factors.

In order to measure intervention effects at follow-up in PA and dietary behavior, multilevel linear regression analysis (MLA) was applied. A two-level data structure was used with individuals (level 1) nested within hospitals (level 2), taking the possible aftercare differences between hospitals into consideration because there might be interdependence between participants from the same hospital. Model testing proceeded in two phases, the “crude” and “adjusted” analyses, in line with Twisk [77]. The “crude model” included the dependent variable (behavior), the intervention condition (0 = UC, 1 = IC), and the baseline value of the outcome behavior as fixed intercepts with random slopes, and hospital as random intercept. All random parameters were added with an independent data structure. Next, the crude model was adjusted for standard demographic and disease-related characteristics, significant variables from dropout analysis, and baseline differences, i.e., gender, age, marital status, education level, income level, employment status, BMI, type of cancer, having had cancer before, type of treatment, time since completion of primary cancer treatment, aftercare, comorbidities, vegetable, fruit, whole grain bread, and fish intake at baseline. These variables were added as fixed intercepts and dummy-coding was used for categorical variables including more than two categories.

For testing the effect of following a specific module, “intervention condition” was categorized into three categories (0 = UC, 1 = IC, specific module not followed, 2 = IC, specific module followed) in the fully adjusted MLA models.

Analyzing the intervention effect on smoking behavior after 6 months by using multilevel logistic regression analysis was not possible due to the small number of smokers. Chi-square tests were applied to assess differences between IC and UC at baseline and follow-up.

Cohen’s *d* effect sizes were calculated for the main effects results on PA and dietary behavior by dividing the difference between the relevant two means of IC en UC at follow-up by the pooled standard deviations of those means [78]. For the sub-analysis of following modules (yes/no), Cohen’s *d* was adjusted for the baseline value by dividing the difference between the means of the relevant change scores by the pooled standard deviation of those means. Additionally, Cohen’s *f*^2^ was calculated in order to evaluate the local effect size within the context of the fully adjusted MLA model with *f*^2^ ≥ 0.02, *f*^2^ ≥ 0.15, and *f*^2^ ≥ 0.35 represent small, medium, and large effect sizes, respectively [78, 79]. To index the magnitude of the effect for smoking, according to Durlak [80], the odds ratios (OR) were calculated by comparing the odds of smoking cessation for the intervention group with the odds of smoking cessation for the control group.

For generating CT messages within the intervention, it was necessary that respondents filled out all questions of the baseline measurement. Consequently, only those respondents, who completed the baseline measurement without missing data, were included in analyses. To assess the intervention effects among respondents who also participated during the follow-up measurement, only complete cases were analyzed. This means that cases with missing data at the follow-up measurement were excluded. Besides that, intention-to-treat analysis (ITT) has been conducted in order to additionally display unbiased estimates of the intervention effects [81]. For PA and dietary behavior outcomes, multiple imputation analyses were conducted by including all variables of the fully adjusted MLA model into the multiple imputation process and using 20 imputed datasets. This is in accordance with the argumentation of Enders [82]. With regard to smoking outcomes, for ITT, participants who were identified as smokers at baseline were accounted as smokers if their smoking status after 6 months could not be determined [83].

By exploring effects on multiple outcomes in dietary behavior and PA, type 1 error might occur due to multiple comparisons. The false discovery rate correcting procedure (FDR) of Benjamini and Hochberg was applied to account for multiple testing problems which is a more powerful procedure as compared to procedures controlling the traditional familywise error rate [84, 85].

## Results

**Fig. 2:**
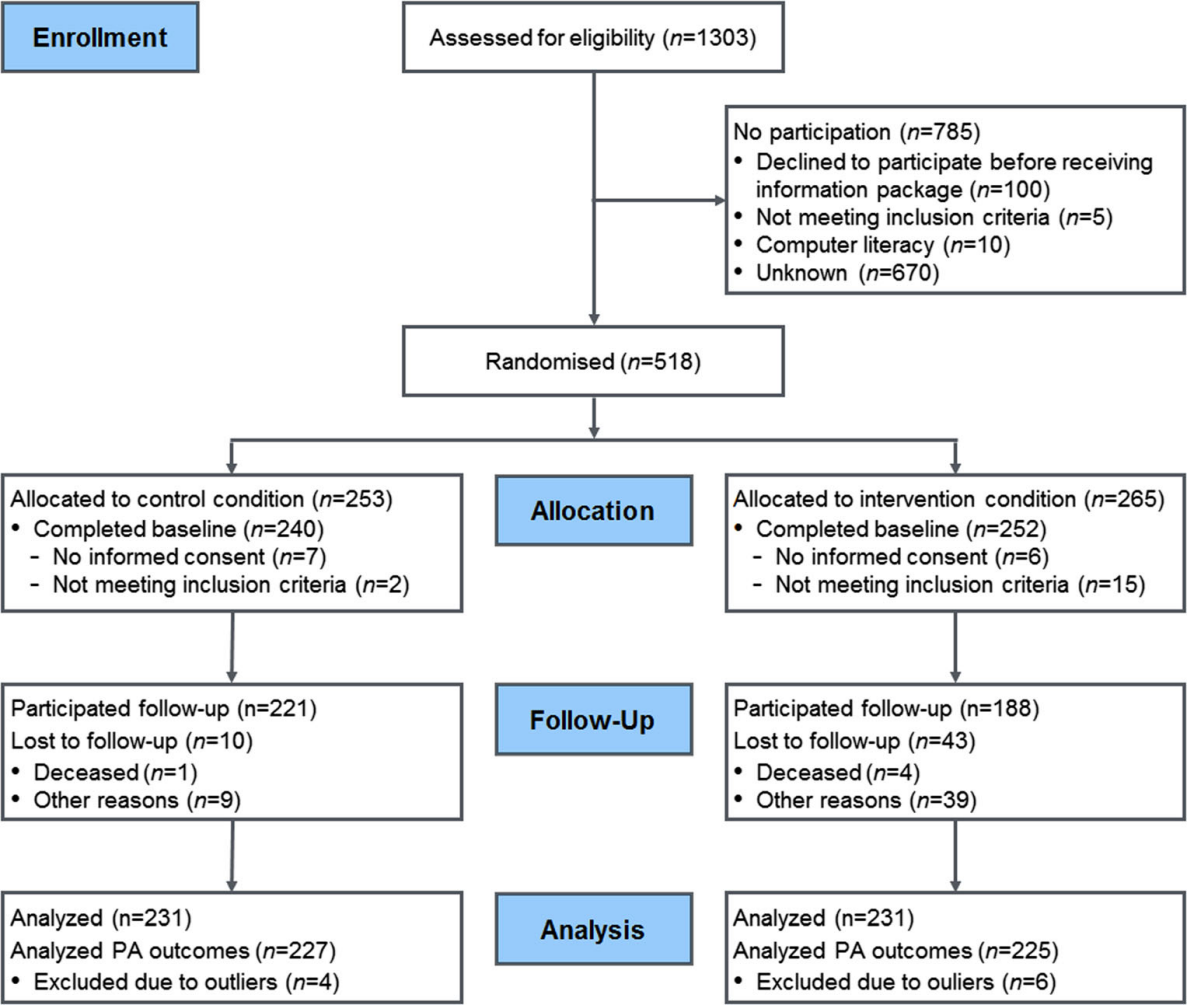
Flow diagram of the reach and attrition of the KNW intervention participants

An overview of the reach and attrition of the intervention participants is provided in Fig. 2. In total, 462 cancer survivors were included for analysis at baseline (IC *n* = 231, UC *n* = 231), and 409 participants filled out the follow-up questionnaire (11.5 % dropout). From the analyses concerning PA outcomes, 10 cases were excluded due to extreme over reporting (>6720 min p/w PA), according to the scorings manual of Wendel-Vos and Schuit, 2004 [66].

The sample characteristics at baseline and lifestyle behavior at baseline and after 6 months are displayed in Table 1. Significant baseline differences between groups were type of treatment, and consumption of vegetable, whole grain bread, and fish. Dropout was higher in the IC (*n* = 43, 18.6 %) than in the UC (*n* = 10, 4.3 %). Significant predictors for dropout were allocation to IC (B = 1.998, SE = .410; *p* = .000), male gender (B = 1.490, SE = .681, *p* = .029), lower modal income (B = 1.155, SE = .513; *p* = .025), lower vegetable consumption (B = −.008, SE = .003; *p* = .014), and higher fruit consumption (B = 0.374, SE = .153; *p* = .014).

**Table 1:** Sample characteristics at baseline, and lifestyle behavior at baseline, and after 6 months

	Baseline			Baseline		After 6 months	
Sample characteristics	Intervention group (*N* = 231)	Control group (*N* = 231)	Lifestyle behavior^a^	Intervention group	Control group	Intervention group	Control group
Female, *n* (%)	183 (79.2)	186 (80.5)	Weekly days > 30 min PA; M (SD)	4.93 (1.87)	4.62 (2.02)	5.11 (1.89)	4.94 (1.84)
Age, M (SD)	55.6 (11.5)	56.2 (11.3)	Change			0.18	0.31
With partner *n* (%)	193 (83.5)	184 (79.7)	Light PA min p/w, M (SD)	1521.46 (897.86)	1430.23 (897.67)	1566.15 (960.78)	1660.62 (992.33)
Resident children, *n* (%)	78 (44.1)	82 (44.6)	Change			44.69	230.39
Low education, *n* (%)	76 (32.9)	97 (42)	Moderate PA min p/w, M (SD)	595.91 (620.50)	525.44 (545.50)	746.64 (676.31)	601.43 (510.90)
Medium education, *n* (%)	76 (32.9)	70 (30.3)	Change			150.73	75.99
High education, *n* (%)	79 (34.2)	64 (27.7)	Vigorous PA min p/w, M (SD)	231.00 (323.88)	238.02 (426.03)	317.95 (458.36)	314.46 (489.92)
Employed, *n* (%)	122 (52.8)	111 (48.1)	Change			86.95	76.44
Not employed, *n* (%)	109 (47.2)	120 (51.9)	Vegetable intake, g p/d M (SD)	138.47 (67.92)	124.17 (57.53)*	146.58 (55.98)	124.92 (60.84)
Income below average, *n* (%)	28 (12.1)	42 (18.2)	Change			8.12	0.75
Income average, *n* (%)	84 (36.4)	78 (33.8)	Fruit intake, servings p/d M (SD)	1.78 (1.23)	1.59 (1.03)	1.87 (0.94)	1.72 (1.08)
Income above average, *n* (%)	119 (51.5)	111 (48.3)	Change			0.10	0.13
Working hours p/w, M (SD)	16.2 (12.0)	16.8 (13.6)	Whole grain bread, slices p/d M (SD)	3.12 (1.81)	2.81 (1.51)*	3.21 (1.48)	2.88 (1.45)
BMI, M (SD)	26.0 (5.0)	26.5 (4.9)	Change			0.09	0.08
Breast cancer, *n* (%)	162 (70.1)	164 (71)	Fish, servings p/w; M (SD)	1.86 (1.93)	1.35 (1.30)*	2.46 (2.75)	1.77 (2.24)
Other cancer type, *n* (%)	69 (29.9)	67 (29)	Change			0.60	0.42
Surgery, chemo, radiation, *n* (%)	86 (37.2)	108 (46.8)*	Smoking behavior total sample				
Surgery, chemo, *n* (%)	61 (26.4)	48 (20.8)	Current smokers	27 (11.7 %)	32 (13.9 %)	18 (10.2 %)	28 (13.5 %)^c^
Surgery, radiation, *n* (%)	46 (19.9)	30 (13)	Change			–9	–4
Other type of treatment, *n* (%)	38 (16.5)	45 (19.5)	Course smoking behavior^b^				
Aftercare, yes, *n* (%)	145 (62.8)	141 (61)	*Complete cases n=50*				
No. aftercare activities, M (SD)	1.1 (1.1)	1 (1.0)	Persistent smokers			18 (81.8 %)	26 (92.9 %)
Comorbidity, yes, *n* (%)	62 (26.8)	63 (27.3)	Quitters			4	2
No. of comorbidities, M (SD)	0.3 (0.6)	0.4 (0.7)	*Intention-to-Treat n = 59*				
Time since primary treatment, No. of weeks, M (SD)	25.1 (13.5)	23.4 (12.9)	Persistent smokers			23 (85.2 %)	30 (93.8 %)
			Quitters			4	2

*BMI* body mass index, *M* mean, *SD* standard deviation, *chemo* chemotherapy, *No* number, *PA* physical activity, *IC* intervention group, *UC* usual care control group, *g* grams

^a^Baseline: PA: IC: *N* = 225; UC: *N* = 227; dietary outcomes: IC: *N* = 231; UC: 231; 6 months follow-up: PA: IC: *N* = 178; UC *N* = 216; dietary outcomes: IC: *N* = 184; UC: 219

^b^Never smokers (IC: *n* = 114, UC: *n* = 109) excluded

^c^Two of the current smokers at 6 months follow-up did not smoke at baseline

*significant result (*p*<0.05)

The IC participants, included into the complete cases analyses, followed on average 2.23 (SD = 1.58) KNW modules. The PA module was followed by 45 (24.73 %), and the module Diet was followed by 116 (61.70 %) of included IC participants. Within the module Diet, 41 (21.81 %) IC participants set a goal to increase their vegetable consumption, 24 (12.77 %) wanted to increase their fruit consumption, 22 (11.7 %) set a goal to increase their fish consumption, 43 (22.87 %) wanted to increase the intake of whole grains, and 10 (5.32 %) set no specific goal. About 80 % followed the module Diet and/or the PA module within 14 weeks after getting access to the KNW. The module Smoking was followed by 19 (10.1 %) of the IC participants included into the complete cases analyses. Almost 95 % of them followed this module within 15 weeks after getting access.

### Physical activity

#### Effects of having access to the KNW on PA after 6 months

As displayed in Table 2, significant differences were found in change over time concerning moderate PA (*B* = 117.738, *p* = .037, *p* fdr = .148, *d* = –0.25, *f*^*2*^ *=* .007) between IC and UC. However, these differences did not remain significant after controlling for multiple testing. No significant intervention effects were found in the other PA outcomes. Their effect sizes ranged from *d* = 0.01 to 0.10; *f*^*2*^ *=* .000 to .006)

**Table 2:** Results from multilevel analysis of the overall intervention effects on physical activity and dietary behavior

	Complete cases analysis^a^						Intention-to-treat analysis^b^			
	B	SE [95 % CI]	*p*	*p* fdr	*d* [95 % CI]	*f* ^*2*^	B	SE [95 % CI]	*p*	*p* fdr
Physical activity outcomes										
Weekly days >30 min										
Crude	.01	0.17 [−.32; .33]	.963	.963			−.08	0.16 [−.40; .23]	.586	.700
Adjusted	.01	0.04 [−.31; .33]	.955	.955	−.10 [−.29; .10]	.000	−.11	0.16 [−.423; .19]	.460	.526
Light PA min p/w										
Crude	−126.47	83.99 [−291.11; 38.16]	.132	.264			−117.85	83.96 [−282.48; 46.79]	.161	.302
Adjusted	−125.59	81.57 [−258.47; 34.29]	.124	.331	.10 [−.10; .30]	.006	−130.57	80.09 [−287.63; 26.49]	.103	.275
Moderate PA min/p/w										
Crude	96.45	51.77 [−5.02; 197.93]	.062	.248			122.18	60.61 [2.82; 241.53]	.045*	.180
Adjusted	117.74	56.45 [7.10; 228.38]	.037*	.148	−.25 [−.45; −.05]	.007	125.24	60.49 [6.06; 244.41]	.040*	.160
Vigorous PA min/p/w										
Crude	−5.31	40.15 [−83.99; 73.38]	.895	.963			2.66	[−77.37; 82.69]	.948	.948
Adjusted	−25.61	38.99 [−102.02; 50.81]	.511	.584	.01 [− .21; .19]	.002	−15.94	[−94.99; 63.11]	.692	.692
Dietary behavior outcomes										
Vegetables (g/p/d)										
Crude	10.26	4.10 [2.23; 18.30]	.012*	.096			11.16	4.19 [2.94; 19.38]	.008	.064
Adjusted	9.15	4.10 [1.03; 17.27]	.027*	.148	−.37 [−.57; −.17]	−.013	9.57	4.21 [1.32; 17.82]	.023*	.160
Fruit (servings p/d)										
Crude	.08	0.07 [−.06; .23]	.242	.323			.07	0.08 [−.08; .22]	.339	.452
Adjusted	.08	0.07 [−.06; .23]	.271	.433	−.15 [−.35; .05]	.002	.07	0.08 [−.08; .22]	.351	.468
Whole grain bread (p/d)										
Crude	.15	0.12 [−.08; .38]	.195	.312			.15	0.12 [−.08; .39]	.189	.302
Adjusted	.11	0.12 [−.12; .34]	.354	.472	−.22 [−.42; −.02]	.000	.11	0.12 [−.12; .34]	.330	.468
Fish (servings p/w)										
Crude	.37	0.23 [−.09; .82]	.113	.264			.34	0.24 [−.13; .82]	.157	.302
Adjusted	.32	0.23 [−.14; .77]	.173	.346	−.28 [−.47; −.08]	.004	.32	0.24 [−.17; .80]	.198	.396

Multilevel analysis with two-level data structure: persons (level1) nested in hospitals (level2); *B* regression coefficient, *d* = Cohen’s *d, f* ^*2*^ = Cohen’s *f* ^*2*^ *. PA* physical activity; *p*/*w* per week, *p*/*d* per day. Crude analysis: models includes intervention condition, outcome behavior at baseline, and hospital; adjusted analysis: adjusted for gender, age, marital status, education level, income level, employment, baseline BMI, cancer type, having had cancer before, treatment type, time since last treatment, participation in aftercare, comorbidities, baseline vegetable, fruit, bread, and fish consumption

^a^For physical activity outcomes *N* = 394; for diet outcomes *N* = 403

^b^ Imputed data: for physical activity outcomes *N* = 452; for diet outcomes *N* = 462

*significant result (*p* < 0.05)

#### Effects of following module PA on PA outcomes

As shown in Table 3, a significant higher increase in moderate PA was found among users of the PA module (*B* = 179.609, *p* = 0.22, *p* fdr = .120, *d* = −0.32, *f*^*2*^ *=* 0.013) compared to participants who did not follow the PA module. This effect did not remain significant after correction for multiple testing.

**Table 3:** Effects of following the behavior-related modules on physical activity and dietary behavior 6 months after getting KNW-access

	B	SE [95 % CI]	d [95 % CI]	*f* ^*2*^	*p*	*p* fdr
Physical activity, UC= ref.						
Numbers of days PA						
Module PA used	.362	.25 [−.14; .86]	−.32 [−.64; .00]	.012	.154	.246
Module PA not used	−.121	.18 [−.47; .22]	.22 [.00; .43]	.002	.492	.656
Light PA						
Module PA used	−119.605	128.82 [−372.08; 132.87]	.13 [−.19; .45]	.006	.353	.403
Module PA not used	127.717	88.90 [−301.95;46.52]	.16 [−.05;.38]	.005	.151	.402
Moderate PA						
Module PA used	197.609	86.09 [28.88; 366.33]	−. 32 [−.64; −00]	.013	.022*	.120
Module PA not used	91.956	61.96 [−29.07; 212.98]	.02 [−.19; 24]	.006	.136	.402
Vigorous PA						
Module PA used	34.531	61.34 [−85.70; 154.76]	−.19 [−.52; .13]	−.000	.573	.573
Module PA not used	−47.259	42.39 [−130.34; 35.82]	.05 [−.16; .27]	.004	.265	.530
Dietary behavior, UC= ref.						
Vegetable consumption						
Module Diet used	7.86	4.81 [−1.55; 17.30]	−.09 [−.31; .14]	−.004	.102	.204
Module Diet not used	11.123	5.62 [.11; 22.14]	−.23 [−.50; .04]	−.018	.048*	.384
Fruit consumption	7.876					
Module Diet used	.181	.08 [.02; .35]	−.12 [−.35; .10]	.016	.031*	.120
Module Diet not used	−.075	.10 [−.27; .12]	.05 [−.22; .32]	.006	.444	.656
Fish consumption						
Module Diet used	.542	.27 [.01; 1.07]	−.11 [−.34; .11]	−.002	.045*	.120
Module Diet not used	−.021	.31 [−.63; .59]	.03 [−.24; .30]	.000	.946	.965
Bread consumption						
Module Diet used	.177	.14 [−.09; .44]	−.02 [−.25; .21]	−.000	.191	.254
Module Diet not used	.001	.16 [−.30; .31]	.03 [−.23 −.31]	.002	.965	.965

Effect of module use was tested using 3 categories: 0 = UC, 1 = IC, specific module not used; 2 = IC, specific module used. Results of the fully adjusted models displayed. Dietary outcomes: *N* = 403, PA outcomes *N* = 394

*IC* intervention condition, *UC* usual care control condition, *ref* reference group, *PA* physical activity; *p* fdr = controlling for false discovery rate; *d*=Cohen’s *d* (corrected for baseline value)*, f* ^*2*^= Cohen’s *f* ^*2*^ *: f* ^*2*^ *≥0.02, f* ^*2*^ *≥0.15, and f* ^*2*^ *.≥0.35* represent small, medium, and large effect sizes

*significant result (*p* < 0.05)

### Diet behavior

#### Effects of having access to the KNW on dietary behavior after 6 months

Significant intervention effects on vegetable consumption using the fully adjusted MLA model (complete cases: *B* = 9.15, *p* = .027, *p* fdr = *.148, d* = −0.37, *f*^*2*^ = −.013; ITT: *B* = 9.57, *p* = .023, *p* fdr = .160) did not remain significant after accounting for multiple testing. No significant effects of having access to the KNW were found on the other dietary behavior outcomes after 6 months. Results are displayed in Table 2.

#### Effects of following module Diet on diet behavior

As displayed in Table 3, users of the module Diet had a significantly higher increase in fruit (*B* = .181, *p* = .031, *p* fdr = .120, *d* = −0.12, *f*^*2*^ = .016) and fish intake (*B* = .542, *p* = .045, *p* fdr = .120 *d* = −0.11, *f*^*2*^ = −.002) after 6 months. A significant increase in vegetable consumption was found among participants who did not follow module Diet (*B* = 11.123, *p* = .048, *p* fdr = .384, *d* = −0.23, *f*^*2*^ = −.018). However, after controlling for multiple comparisons, these results did not remain significant (Table 3b).

### Smoking behavior after 6 months

At baseline, 27 (11.7 %) respondents of the IC, and 32 (13.9 %) respondents of the UC were current smokers (Table 1). After 6 months, respectively, 18 (7.8 %) and 28 (13.5 %) respondents of IC and UC were current smokers. From the smokers at baseline, 18 (81.8 %) were persistent smokers and 4 (18.8 %) were quitters after 6 months in the IC. In the UC, 26 (92.9 %) were persistent smokers and 2 (7.1 %) were quitters after 6 months. No significant intervention effect was found between groups at follow-up (*X*^2^ = 1.42, *p* = .233, OR 2.89). ITT revealed comparable results (*X*^2^ = 1.18, *p* = .278, OR = 2.61) (*X*^*2*^ tests are not displayed in Table 1).

## Discussion

The present RCT evaluated the effects of the web-based, CT, multiple behavior KNW intervention on lifestyle outcomes, i.e., PA, diet (vegetable, fruit, whole grain bread, and fish consumption), and smoking behavior after 6 months. The presented outcomes point in the direction that the KNW may affect moderate PA and dietary behaviors. Cancer survivors who had access to the KNW showed larger increases in moderate PA and vegetable consumption, and using the specific modules resulted in a larger increase of moderate PA, and larger increases in fruit and fish consumption. These effects need to be interpreted with caution, however, since results did not remain significant after correction for multiple testing.

The loss to follow-up after 6 months was low (11.5 %) in comparison with the mean percentage of dropouts (19.7 %) of web-based trials for cancer survivors [54]. This suggests a strong commitment that may be attributed to an evident need of cancer survivors for support after completion of primary cancer treatment [21]. This period can be considered as a teachable moment. Additionally, low dropout in the UC after 6 months (Fig. 2) suggests that allocation to the waiting list UC was well accepted by the participants.

### Physical Activity

The increase in moderate PA in the IC that was found in the main analysis (having KNW access) was confirmed when testing the use of the specific PA module. The effect size of moderate PA changes (*d* = 0.32) was higher when testing the use of the PA module compared to the main analysis (*d* = 0.25). In comparison with prior, web-based PA-only interventions, these effect sizes were similar or even higher than the earlier reported overall effect size of *d* = 0.14 [86]. Possibly, the module PA was followed by cancer survivors who were actually “in need” and able to increase PA. This might indicate that the KNW advice on PA could have targeted the desired subpopulation. The low number of module PA followers (*n* = 45) might possibly have caused power problems, which might be a reason for the non-significant results after controlling for multiple testing. Moreover, the raw data (Table 1) of increases in moderate PA (+150.73 min p/w) in the IC was notably higher as compared to the UC (+75.99 min p/w). This may be interpreted as a meaningful result, considering findings from Wen et al. [87] that every additional 15 min a day or 90 min a week of moderately intense PA reduced all-cancer mortality. This dose-response relationship has been confirmed in recent publications [3, 88, 89].

The PA module was derived from an existing, basic web-based PA intervention for the general population aged over 50, named Active Plus [38, 41], which has been shown to be effective in increasing weekly minutes of moderate and vigorous PA after 6 months (*d* = .24). The increase of combined moderate and vigorous PA was higher in the Active Plus intervention in comparison to the KNW intervention (283 min vs. 238 min p/w). Reasons for these differences might be the target population (general population in Active Plus vs cancer survivors in KNW) and the program intensity (three tailored sessions within 6 months in Active Plus vs one combined tailored session followed by an evaluation 4 weeks later in KNW). In addition, the PA module was one of eight modules in the KNW, while the Active Plus intervention consisted of only the theme on PA. In addition, there might have been more selective attrition in Active Plus due to higher dropout (close to half of the sample).

### Dietary behavior

The effects of the KNW on dietary behavior are valuable to mention although they remained not significant after accounting for multiple testing. It is promising that participants who had access to the KNW showed a higher vegetable consumption. As can be concluded from the sub-analyses, this increase in vegetable consumption could not be explained by following the module Diet. Possibly, the increase in vegetable consumption might be attributed to other intervention components, such as one of the news items that targeted the topic diet very extensively and which were distributed to all IC participants. The module Diet was followed by more than 60 % of the participants, which suggests that this module was popular, and possibly not only visited based on the provided advice, but also based on self-selection. Those who followed the module Diet had a higher increase in fruit and fish consumption. A possible explanation for the effect on fish consumption might be attributed to an increase in knowledge about the health advantages of consuming fatty fish, and that consuming fatty fish twice a week may be a healthier choice than eating red and processed meat on daily basis. With regard to the effect on fruit consumption, higher increases in fruit consumption on daily basis might be easier to achieve than changes in other diet habits. Furthermore, it was not possible to choose more than two goals within the module Diet, which resulted in the lower numbers of participants who set goals on the specific dietary outcomes. This might be an explanation for the non-significant results after correction for multiple testing.

The effect size for changes in vegetable consumption (*d* = 0.37) in the present study was in line with the effect size of a Dutch web-based, CT, diet-only education intervention for adults in the general population (*d* = 0.32) [90]. Also, Goode et al. [55] reported comparable effect sizes (*d* = 0.16 to *d* = 1.71) for non-face-to-face interventions on fruit and vegetable outcomes. Most of these reported studies included intensive (telephone) counseling for cancer survivors. In contrast, the module Diet included less separate sessions; however, it showed comparable outcomes. In addition, the web-based multiple behavior intervention for cancer survivors reported by Bantum [56] was not effective in changing dietary behavior, although not accounting for multiple testing. Parsons et al. [91] also reported significant changes in vegetable consumption, but not in other dietary behaviors, 6 months after diet telephone counseling among prostate cancer patients.

Notably, the average consumption of vegetables, fruit, whole grain bread, and fish were below recommended levels among the whole sample at both time points. These results confirm findings from research among Dutch cancer survivors, reporting that only 27.4 % has met the vegetable recommendations [17]. Additionally, a low overall intake of healthy food has been reported in several studies [6, 12, 16]. At the same time, recent observations revealed that particularly early cancer survivors were more likely to meet the vegetable and fruit recommendations [13, 92]. Still, as our results suggest, there is a lot of room for improvement in dietary behaviors among cancer survivors, and intervening shortly after completing primary treatment seems to be a very relevant period and apparently a teachable moment.

### Smoking Behavior

No significant intervention effects have been found for smoking behavior after 6 months. However, the likelihood of giving up smoking was almost three times higher in the IC than in the UC (OR = 2.89). Nonetheless, this has to be interpreted with caution due to the limited amount of smokers in our study population. With higher numbers of smokers and the possibility to apply multilevel logistic regression analysis, it could be expected that significant results might occur in favor of the IC.

### Multiple behavior interventions

This multiple behavior KNW intervention was especially designed to cover a broad range of relevant topics to meet the various cancer survivors’ needs [17, 21]. Besides targeting PA, diet, and smoking, the KNW also offered modules targeting fatigue, anxiety and depression, relational problems, return to work, and residual problems. The lifestyle modules included less separate sessions as compared to other multiple health behavior change interventions for cancer survivors [55, 56, 93]. This could be a possible reason for the limited effects of the KNW on lifestyle. Moreover, it might have been difficult for cancer survivors to focus on numerous topics. Most of the participants visited two modules, and possibly, for some of the participants the psychosocial topics had a higher priority. Earlier research revealed that in the first year after cancer treatment, residual and psychosocial problems might impede lifestyle change [92]. Furthermore, within the KNW, the number of recommendations to follow a certain module varied individually with a broad range from zero to eight. This was dependent on the responses given at baseline. Wilson et al. [94] described that intervention effects might be curvilinear related to the number of recommendations given, with a moderate number of recommendation being most beneficial among the general population.

### Limitations

This RCT provided insightful and valuable findings despite the limited effects on lifestyle behaviors. Nevertheless, some limitations should be acknowledged. Regarding generalizability, the KNW participants were mainly middle-aged breast cancer survivors with an above average income level and without comorbidities. This might be too selective to represent the general cancer survivor population. However, these findings are in line with the prevalence of breast cancer in the Netherlands [95] and with Kohl et al. [33], confirming a higher reach of web-based interventions among female participants with higher socioeconomic status. Furthermore, the intervention tested is an eHealth intervention and participation demanded that participants had internet access and sufficient computer skills. These intervention characteristics can also explain the overrepresentation of participants who are younger and generally more highly educated.

Present results might have been influenced by the selective dropout. However, the dropout rate was very low, analyses were corrected for the corresponding variables, and intention-to-treat analyses revealed comparable results to complete cases analyses. Besides this, health behaviors were measured using self-report questionnaires, thus allowing over- and underestimations to occur due to social desirability or recall bias [96]. Although the self-administrated questionnaires were validated, easy to apply, inexpensive, and have often been used in large-scale studies, we may presume that overestimation occurred in PA [39, 65, 67, 72, 74, 97, 98]. The proportion of smoking cessation might be slightly underestimated due to accounting smokers at baseline as smokers in intention-to-treat-analyses if their smoking behavior could not be measured after 6 months.

Prior to the baseline assessment, the participants knew about their group assignment, which might have influenced the responses on the baseline questionnaire. We assume, however, that the baseline differences in dietary behavior occurred merely by chance, given the comparable response of participants in both intervention conditions at baseline. There were also no differences in PA and smoking behavior at baseline. In addition, in this RCT, the intervention was compared to a usual-care control group, who possibly participated in other aftercare interventions. Multilevel linear regression analysis was applied for addressing possible differences in (after-) care between the different hospitals, and all analyses were corrected for aftercare use.

## Conclusion

Having access to the KNW and following the KNW modules do affect lifestyle behaviors, although to a limited extent. Meaningful increases in moderate PA were detected in the IC, and the effect size of the increase in vegetable consumption was higher than in comparable studies. Moreover, the outcomes point in the direction that following the module Diet could affect fruit and fish consumption. Non-significant results after accounting for multiple testing in moderate PA, vegetable, fruit and fish consumption might be due to the high number of outcomes and the low numbers of module users who set a goal on the specific outcome behavior. No significant intervention effect was found on smoking behavior due to the low number of smokers. An exploration of the use of this complex KNW intervention is recommended to get further insights into underlying mechanisms and to improve the intervention effectiveness. Overall, results provide preliminary indications that this theory-based, wide-ranging web-based cancer aftercare intervention can provide valuable support in usual cancer aftercare.

## Footnotes

1 After recruitment, it was observed that participants had been included slightly earlier or later than initially defined (6–52 weeks after completion of primary treatment) [41]. This inclusion criterion was broadened to enable analysis of data filled out 4 weeks (*N* = 13) and up to 56 weeks (*N* = 7) after completion of primary treatment. We assumed that cancer survivors, who voluntarily participated 2 weeks earlier and 1 month later than predetermined, were not different as compared to the other participants.

## References

[1] Baena R, Salinas P (2015). Diet and colorectal cancer. Maturitas.

[2] Baena Ruiz R, Salinas Hernandez P (2013). Diet and cancer: risk factors and epidemiological evidence. Maturitas.

[3] Schmid D, Leitzmann MF (2014). Association between physical activity and mortality among breast cancer and colorectal cancer survivors: a systematic review and meta-analysis. Ann Oncol.

[4] Husson O, Mols F, Ezendam NPM, Schep G, van de Poll-Franse LV (2015). Health-related quality of life is associated with physical activity levels among colorectal cancer survivors: a longitudinal, 3-year study of the PROFILES registry. J Cancer Surviv.

[5] Florou AN, Gkiozos IC, Tsagouli SK, Souliotis KN, Syrigos KN (2014). Clinical significance of smoking cessation in subjects with cancer: a 30-year review. Respir Care.

[6] Vijayvergia N, Denlinger CS (2015). Lifestyle factors in cancer survivorship: where we are and where we are headed. J Personalized Med.

[7] Rock CL, Doyle C, Demark-Wahnefried W, Meyerhardt J, Courneya KS, Schwartz AL (2012). Nutrition and physical activity guidelines for cancer survivors. CA Cancer J Clin.

[8] Research WCRFAIfC (2009). Policy and Action for Cancer Prevention. Food, Nutrition, and Physical Activity: a Global Perspective.

[9] Kushi LH, Doyle C, McCullough M, Rock CL, Demark-Wahnefried W, Bandera EV (2012). American Cancer Society Guidelines on nutrition and physical activity for cancer prevention: reducing the risk of cancer with healthy food choices and physical activity. CA Cancer J Clin.

[10] Network NCC. NCCN Clinical Practice Guidelines in Oncology (NCCN Guidelines). Smoking Cessation. Version 1.2015..2015. http://www.nccn.org/professionals/physician_gls/f_guidelines.asp#smoking_cessation. Accessed 07.10.2015.

[11] Boyle JM, Tandberg DJ, Chino JP, D’Amico TA, Ready NE, Kelsey CR (2015). Smoking history predicts for increased risk of second primary lung cancer: a comprehensive analysis. Cancer.

[12] Blanchard CM, Courneya KS, Stein K (2008). American Cancer Society’s SCS, II. Cancer survivors’ adherence to lifestyle behavior recommendations and associations with health-related quality of life: results from the American Cancer Society’s SCS-II. J Clin Oncol.

[13] LeMasters TJ, Madhavan SS, Sambamoorthi U, Kurian S (2014). Health behaviors among breast, prostate, and colorectal cancer survivors: a US population-based case-control study, with comparisons by cancer type and gender. J Cancer Surviv.

[14] Westmaas JL, Alcaraz KI, Berg CJ, Stein KD (2014). Prevalence and correlates of smoking and cessation-related behavior among survivors of ten cancers: findings from a nationwide survey nine years after diagnosis. Cancer Epidemiol Biomarkers Prev.

[15] Inoue-Choi M, Robien K, Lazovich D (2013). Adherence to the WCRF/AICR guidelines for cancer prevention is associated with lower mortality among older female cancer survivors. Cancer Epidemiol Biomarkers Prev.

[16] Zhang FF, Liu S, John EM, Must A, Demark-Wahnefried W. Diet quality of cancer survivors and noncancer individuals: Results from a national survey. Cancer. 2015. doi:10.1002/cncr.29488. 10.1002/cncr.29488 PMC4667562 26624564

[17] Kanera IM, Bolman CA, Mesters I, Willems RA, Beaulen AA, Lechner L (2016). Prevalence and correlates of healthy lifestyle behaviors among early cancer survivors. BMC Cancer.

[18] James-Martin G, Koczwara B, Smith EL, Miller MD (2014). Information needs of cancer patients and survivors regarding diet, exercise and weight management: a qualitative study. Eur J Cancer Care.

[19] Kwok A, Palermo C, Boltong A (2015). Dietary experiences and support needs of women who gain weight following chemotherapy for breast cancer. Support Care Cancer.

[20] Pullar JM, Chisholm A, Jackson C (2012). Dietary information for colorectal cancer survivors: an unmet need. N Z Med J.

[21] Willems RA, Bolman CA, Mesters I, Kanera IM, Beaulen AA, Lechner L (2015). Cancer survivors in the first year after treatment: the prevalence and correlates of unmet needs in different domains. Psychooncology.

[22] Anderson AS, Caswell S, Wells M, Steele RJ (2013). Obesity and lifestyle advice in colorectal cancer survivors—how well are clinicians prepared?. Colorectal Dis.

[23] Wu HS, Harden JK (2015). Symptom burden and quality of life in survivorship: a review of the literature. Cancer Nurs.

[24] Coa KI, Smith KC, Klassen AC, Caulfield LE, Helzlsouer K, Peairs K (2015). Capitalizing on the "teachable moment" to promote healthy dietary changes among cancer survivors: the perspectives of health care providers. Support Care Cancer.

[25] Warren GW, Marshall JR, Cummings KM, Toll B, Gritz ER, Hutson A (2013). Practice patterns and perceptions of thoracic oncology providers on tobacco use and cessation in cancer patients. J Thorac Oncol.

[26] Demark-Wahnefried W, Rogers LQ, Alfano CM, Thomson CA, Courneya KS, Meyerhardt JA (2015). Practical clinical interventions for diet, physical activity, and weight control in cancer survivors. CA Cancer J Clin.

[27] De Angelis R, Sant M, Coleman MP, Francisci S, Baili P, Pierannunzio D et al. Cancer survival in Europe 1999–2007 by country and age: results of EUROCARE-5—a population-based study. Lancet Oncol. 2014;15(1):23–34. doi:10.1016/S1470-2045(13)70546-1. 10.1016/S1470-2045(13)70546-1 24314615

[28] Hammer MJ, Ercolano EA, Wright F, Dickson VV, Chyun D, Melkus GD (2015). Self-management for adult patients with cancer: an integrative review. Cancer Nurs.

[29] Integraal Kankercentrum Nederland (Comprehensive Cancer Centre the Netherlands I. Guideline Cancer survivorship care. 2011. http://www.oncoline.nl/index.php?language=en.

[30] Azadmanjir Z, Safdari R, Ghazisaeidi M (2015). From self-care for healthy people to self-management for cancer patients with cancer portals. Asian Pac J Cancer Prev.

[31] Chou WY, Liu B, Post S, Hesse B (2011). Health-related Internet use among cancer survivors: data from the Health Information National Trends Survey, 2003-2008. J Cancer Surviv.

[32] Warren E, Footman K, Tinelli M, McKee M, Knai C (2014). Do cancer-specific websites meet patient’s information needs?. Patient Educ Couns.

[33] Kohl LF, Crutzen R, de Vries NK (2013). Online prevention aimed at lifestyle behaviors: a systematic review of reviews. J Med Internet Res.

[34] Neville LM, O’Hara B, Milat A (2009). Computer-tailored physical activity behavior change interventions targeting adults: a systematic review. Int J Behav Nutr Phys Act.

[35] Schulz DN, Kremers SP, Vandelanotte C, van Adrichem MJ, Schneider F, Candel MJ (2014). Effects of a Web-based tailored multiple-lifestyle intervention for adults: a two-year randomized controlled trial comparing sequential and simultaneous delivery modes. J Med Internet Res.

[36] Broekhuizen K, Kroeze W, van Poppel MN, Oenema A, Brug J (2012). A systematic review of randomized controlled trials on the effectiveness of computer-tailored physical activity and dietary behavior promotion programs: an update. Ann Behav Med.

[37] van Stralen MM, de Vries H, Mudde AN, Bolman C, Lechner L (2009). Efficacy of two tailored interventions promoting physical activity in older adults. Am J Prev Med.

[38] Peels DA, van Stralen MM, Bolman C, Golsteijn RH, de Vries H, Mudde AN (2014). The differentiated effectiveness of a printed versus a Web-based tailored physical activity intervention among adults aged over 50. Health Educ Res.

[39] Te Poel F, Bolman C, Reubsaet A, de Vries H (2009). Efficacy of a single computer-tailored e-mail for smoking cessation: results after 6 months. Health Educ Res.

[40] Civljak M, Stead LF, Hartmann-Boyce J, Sheikh A, Car J (2013). Internet-based interventions for smoking cessation. Cochrane Database Syst Rev.

[41] Willems RA, Bolman CA, Mesters I, Kanera IM, Beaulen AA, Lechner L (2015). The Kanker Nazorg Wijzer (Cancer Aftercare Guide) protocol: the systematic development of a Web-based computer tailored intervention providing psychosocial and lifestyle support for cancer survivors. BMC Cancer.

[42] de Vries H, Mudde A, Leijs I, Charlton A, Vartiainen E, Buijs G (2003). The European Smoking Prevention Framework Approach (EFSA): an example of integral prevention. Health Educ Res.

[43] Fishbein M, Ajzen I (2010). Predicting and changing behavior. the reasoned action approach.

[44] Ajzen I (2011). The theory of planned behaviour: reactions and reflections. Psychol Health.

[45] Baumeister RF, Heatherton TF, Tice DM (1994). Losing control: how and why people fail at self-regulation.

[46] Stacey FG, James EL, Chapman K, Courneya KS, Lubans DR (2015). A systematic review and meta-analysis of social cognitive theory-based physical activity and/or nutrition behavior change interventions for cancer survivors. J Cancer Surviv.

[47] Green HJ, Steinnagel G, Morris C, Laakso EL (2014). Health behaviour models and patient preferences regarding nutrition and physical activity after breast or prostate cancer diagnosis. Eur J Cancer Care.

[48] de Vries H, Eggers SM, Bolman C (2013). The role of action planning and plan enactment for smoking cessation. BMC Public Health.

[49] Peels D. Promoting physical activity of people aged over fifty. Feasibility and (cost-)effectiveness of the Web-based versus the print-delivered computer tailored Activ Plus intervention. Heerlen, The Netherlands: 2014.

[50] Bolman C, Eggers SM, van Osch L, Te Poel F, Candel M, de Vries H. Is Action Planning Helpful for Smoking Cessation? Assessing the Effects of Action Planning in a Web-Based Computer-Tailored Intervention. Substance use & misuse. 2015:1-12. doi:10.3109/10826084.2014.977397. 10.3109/10826084.2014.977397 26440754

[51] Lechner LM, I. Bolman, C.A.W. Gezondheidspsychologie bij patiënten. 1st ed. Assen: Koninklijke Van Gorcum BV; 2010.

[52] Bartholomew LK, Parcel GS, Kok G, Gottlieb NH, Fernandez ME. Planning Health Promotion Programs. An Intervention Mapping Approach. 3rd ed. San Francisco: Jossey-Bass; 2011.

[53] Noar SM, Benac CN, Harris MS (2007). Does tailoring matter? Meta-analytic review of tailored print health behavior change interventions. Psychol Bull.

[54] Kuijpers W, Groen WG, Aaronson NK, van Harten WH (2013). A systematic review of Web-based interventions for patient empowerment and physical activity in chronic diseases: relevance for cancer survivors. J Med Internet Res.

[55] Goode AD, Lawler SP, Brakenridge CL, Reeves MM, Eakin EG (2015). Telephone, print, and Web-based interventions for physical activity, diet, and weight control among cancer survivors: a systematic review. J Cancer Surviv.

[56] Bantum EO, Albright CL, White KK, Berenberg JL, Layi G, Ritter PL (2014). Surviving and thriving with cancer using a Web-based health behavior change intervention: randomized controlled trial. J Med Internet Res.

[57] Lee MK, Yun YH, Park HA, Lee ES, Jung KH, Noh DY (2014). A Web-based self-management exercise and diet intervention for breast cancer survivors: pilot randomized controlled trial. Int J Nurs Stud.

[58] De Cocker K, Charlier C, Van Hoof E, Pauwels E, Lechner L, Bourgois J (2014). Development and usability of a computer-tailored pedometer-based physical activity advice for breast cancer survivors. Eur J Cancer Care.

[59] Emmons KM, Puleo E, Sprunck-Harrild K, Ford J, Ostroff JS, Hodgson D (2013). Partnership for health-2, a Web-based versus print smoking cessation intervention for childhood and young adult cancer survivors: randomized comparative effectiveness study. J Med Internet Res.

[60] Systems. OSEHP. TailorBuilder. http://www.ose.nl/nl/tailorbuilder.html.

[61] Ministerie van Volksgezondheid WeS. Medisch-wetenschappelijk onderzoek. Algemene informatie voor proefpersonen. 2014.

[62] de Vries H, Brug J (1999). Computer-tailored interventions motivating people to adopt health promoting behaviours: introduction to a new approach. Patient Educ Couns.

[63] Davies NJ, Batehup L, Thomas R (2011). The role of diet and physical activity in breast, colorectal, and prostate cancer survivorship: a review of the literature. Br J Cancer.

[64] D’Zurilla TJ, Nezu AM (2007). Problem-solving therapy: a positive approach to clinical intervention.

[65] Wendel-Vos GCW, Schuit AJ, Saris WHM, Kromhout D (2003). Reproducibility and relative validity of the short questionnaire to assess health-enhancing physical activity. J Clin Epidemiol.

[66] Wendel-Vos W. SJ. SQUASH Short QUestionnaire to ASses Health enhancing physical activity: Centrum voor Preventie en Zorgonderzoek Rijksinstituut voor Volksgezondheid en Milieu; 2004.

[67] de Hollander EL, Zwart L, de Vries SI, Wendel-Vos W (2012). The SQUASH was a more valid tool than the OBiN for categorizing adults according to the Dutch physical activity and the combined guideline. J Clin Epidemiol.

[68] Milton K, Bull FC, Bauman A (2011). Reliability and validity testing of a single-item physical activity measure. Br J Sports Med.

[69] Milton K, Clemes S, Bull F (2013). Can a single question provide an accurate measure of physical activity?. Br J Sports Med.

[70] Wagenmakers R, van den Akker-Scheek I, Groothoff JW, Zijlstra W, Bulstra SK, Kootstra JW (2008). Reliability and validity of the short questionnaire to assess health-enhancing physical activity (SQUASH) in patients after total hip arthroplasty. BMC Musculoskelet Disord.

[71] Arends S, Hofman M, Kamsma YP, van der Veer E, Houtman PM, Kallenberg CG (2013). Daily physical activity in ankylosing spondylitis: validity and reliability of the IPAQ and SQUASH and the relation with clinical assessments. Arthritis Res Ther.

[72] Brink CL van den OM, Houben AW, Nierop P van, Droomers M. Validering van standaardvraagstelling voeding voor Lokale en Nationale Monitor Volksgezondheid RIVM 2005. Report No.: 260854008.

[73] Bogers RP, Van Assema P, Kester AD, Westerterp KR, Dagnelie PC (2004). Reproducibility, validity, and responsiveness to change of a short questionnaire for measuring fruit and vegetable intake. Am J Epidemiol.

[74] Mudde AN, Willemsen MC, Kremers S, De Vries H (2006). Meetinstrumenten voor onderzoek naar roken en stoppen met roken.

[75] Hughes JR, Keely JP, Niaura RS, Ossip-Klein DJ, Richmond RL, Swan GE (2003). Measures of abstinence in clinical trials: issues and recommendations. Nicotine Tob Res.

[76] Velicer WF, Prochaska JO (2004). A comparison of four self-report smoking cessation outcome measures. Addict Behav.

[77] Twisk J (2006). Applied multilevel analysis. A Practical Guide.Practical Guides to Biostatistics and Epidemiology.

[78] Cohen J (1992). A power primer. Psychol Bull.

[79] Selya AS, Rose JS, Dierker LC, Hedeker D, Mermelstein RJ (2012). A practical guide to calculating Cohen’s f(2), a measure of local effect size, from PROC MIXED. Front Psychol.

[80] Durlak JA (2009). How to select, calculate, and interpret effect sizes. J Pediatr Psychol.

[81] Montori VM, Guyatt GH (2001). Intention-to-treat principle. CMAJ.

[82] Enders CK. Applied Missing Data Analysis. New York: The Guilford Press; 2010.

[83] West R, Hajek P, Stead L, Stapleton J (2005). Outcome criteria in smoking cessation trials: proposal for a common standard. Addiction.

[84] Benjamini Y HY. Controlling the false discovery rate: a practical and powerful approach to multiple testing. J Royal Stat Soc Ser. 1995; 57:289–300.

[85] Benjamini YYD (2001). The control of the false discovery rate in multiple testing under dependency. Ann Statist.

[86] Davies CA, Spence JC, Vandelanotte C, Caperchione CM, Mummery WK (2012). Meta-analysis of internet-delivered interventions to increase physical activity levels. Int J Behav Nutr Phys Act.

[87] Wen CP, Wai JP, Tsai MK, Yang YC, Cheng TY, Lee MC, et al. Minimum amount of physical activity for reduced mortality and extended life expectancy: a prospective cohort study. Lancet (London, England). 2011;378(9798):1244-53. doi:10.1016/s0140-6736(11)60749-6. 10.1016/S0140-6736(11)60749-6 21846575

[88] Arem H, Moore SC, Patel A, Hartge P, Berrington de Gonzalez A, Visvanathan K (2015). Leisure time physical activity and mortality: a detailed pooled analysis of the dose-response relationship. JAMA Int Med.

[89] Samitz G, Egger M, Zwahlen M (2011). Domains of physical activity and all-cause mortality: systematic review and dose-response meta-analysis of cohort studies. Int J Epidemiol.

[90] Springvloet L, Lechner L, de Vries H, Oenema A (2015). Long-term efficacy of a Web-based computer-tailored nutrition education intervention for adults including cognitive and environmental feedback: a randomized controlled trial. BMC Public Health.

[91] Parsons JK, Newman VA, Mohler JL, Pierce JP, Flatt S, Marshall J (2008). Dietary modification in patients with prostate cancer on active surveillance: a randomized, multicentre feasibility study. BJU Int.

[92] Bluethmann SM, Basen-Engquist K, Vernon SW, Cox M, Gabriel KP, Stansberry SA (2015). Grasping the ’teachable moment’: time since diagnosis, symptom burden and health behaviors in breast, colorectal and prostate cancer survivors. Psychooncology.

[93] Green AC, Hayman LL, Cooley ME (2015). Multiple health behavior change in adults with or at risk for cancer: a systematic review. Am J Health Behav.

[94] Wilson K, Senay I, Durantini M, Sanchez F, Hennessy M, Spring B (2015). When it comes to lifestyle recommendations, more is sometimes less: a meta-analysis of theoretical assumptions underlying the effectiveness of interventions promoting multiple behavior domain change. Psychol Bull.

[95] Nederland IK. Cijfers over kanker. In: Nederlandse Kankerregistratie. http://www.cijfersoverkanker.nl/.

[96] Van Assema P, Brug J, Ronda G, Steenhuis I, Oenema A (2002). A short dutch questionnaire to measure fruit and vegetable intake: relative validity among adults and adolescents. Nutr Health.

[97] Campbell N, Gaston A, Gray C, Rush E, Maddison R, Prapavessis H (2015). The Short QUestionnaire to ASsess Health-enhancing (SQUASH) physical activity in adolescents: a validation using doubly labeled water. J Phys Act Health.

[98] Helmerhorst HJ, Brage S, Warren J, Besson H, Ekelund U (2012). A systematic review of reliability and objective criterion-related validity of physical activity questionnaires. Int J Behav Nutr Phys Act.

